# Supplementary material for: Time to care: why the humanities and the social sciences belong in the science of health
Source: BMJ Open. 2019 Aug 27;9(8):e030286. doi: 10.1136/bmjopen-2019-030286 (PMC6720150; doi:10.1136/bmjopen-2019-030286)
Supplement: Author's manuscript [file bmjopen-2019-030286.draft_revisions.pdf]

BMJ Open

BMJ Open is committed to open peer review. As part of this commitment we make the peer review history of every article we publish publicly available.

When an article is published we post the peer reviewers' comments and the authors' responses online. We also post the versions of the paper that were used during peer review. These are the versions that the peer review comments apply to.

The versions of the paper that follow are the versions that were submitted during the peer review process. They are not the versions of record or the final published versions. They should not be cited or distributed as the published version of this manuscript.

BMJ Open is an open access journal and the full, final, typeset and author-corrected version of record of the manuscript is available on our site with no access controls, subscription charges or pay-per-view fees (http://bmjopen.bmj.com).

If you have any questions on BMJ Open's open peer review process please email
info.bmjopen@bmj.com

Time to care: why the humanities and the social sciences belong in the science of health

Journal:	*BMJ Open*
Manuscript ID	bmjopen-2019-030286.R1
Article Type:	Communication
Date Submitted by the Author:	27-Mar-2019
Complete List of Authors:	Clarke, B; Imperial College London,<br>Ghiara, Virginia; University of Kent<br>Russo, Federica; Universiteit van Amsterdam,
**Primary Subject Heading**:	Public health
Secondary Subject Heading:	Epidemiology, Global health, Health policy, Qualitative research, Sociology
Keywords:	Medical humanities, Sociology of health, Prevention, Interdisciplinarity

SCHOLARONE™
Manuscripts

Time to care: why the humanities and the social sciences belong in the science of health

A. Authors: Brendan Clarke; Virginia Ghiara; Federica Russo (and co-signatories listed at the end of the article)
B. Corresponding author: Federica Russo, Department of Philosophy, University of Amsterdam, Postbus 94201, 1090 GE Amsterdam, Tel +31 (0)20 525 3537, email: f.russo@uva.nl / federica.russo@gmail.com
C. Co-authors: Brendan Clarke, University College London, London, UK; Virginia Ghiara, University of Kent, Canterbury, UK
D. Keywords: Medical humanities; Sociology of health; Prevention; Interdisciplinarity
E. Word count: 1550

For peer review only

1. Our vision

Health is *more* than a biological phenomenon. It is also inherently social, psychological, cultural, and historical. While this has been recognised by major health actors for decades, open questions remain as to how to build systems that reflect the complexity of health, disease, and sickness, and in a context that is increasingly technologized. Although we find in the literature an increasing understanding that health is more than a biological phenomenon (Blue, Shove, Carmona, & Kelly, 2016; Friel & Marmot, 2011; Gibbon, Prainsack, Hilgartner, & Lamoreaux, 2018; Opolski & Wysocki, 2013), the implementation of this knowledge lags behind. Biological approaches to health and disease, as a matter of fact, dominate the development of curative and preventive interventions.

We argue that an urgent change of approach is necessary. Methods and concepts from the humanities and social science must be *embedded* in the concepts and methods of the health sciences and of public health, if we are to promote sustainable interventions capable of engaging with the recognized complexity of health, disease, and sickness. This resonates with the vision expressed by UK Health Secretary and by many policy documents (Forman et al., 2018; Napier et al., 2014) from the last decades. Yet integrated strategies to

For peer review only - http://bmjopen.bmj.com/site/about/guidelines.xhtml

understand and to intervene on the complexity of health and that engage with biological, social, psychological and behavioural factors are still needed.

Our vision is one of *radical interdisciplinarity*, integrating aspects of biological, psychological, social, and humanities approaches across areas of urgent health need. These areas include, but is not confined to, chronic diseases such as the obesity epidemic, cancer, mental health. Radical interdisciplinarity entails the **practical**, **methodological**, and **conceptual** integration of approaches to health, as they are developed in the health and social sciences, and in the humanities. In what follows, we describe our current understanding of these three aspects, and describe how radical interdisciplinarity would change them.

Practical

How things stand

The impact of social factors on health is widely acknowledged in the scientific literature (Braveman, Egerter, & Williams, 2011; Commission on Social Determinants of Health, 2008; Jia, Sankoh, & Tatem, 2015; Marmot, 2005; Muntaner, 2013). However, this does not always mean that they are effectively actioned in health care and public health. Best practices do exist, but they do not constitute, as yet, the dominant approach. We think that exemplary public health programmes, such as *WHO Tailoring Immunization Programmes*, are strengthened and effective precisely because they explicitly target social factors and exploit deep knowledge of cultural factors and social dynamic in the design of interventions. Despite the successes of interventions targeting social and cultural factors, more distinctively biological models of health predominate in practice (Napier et al., 2014). The dominant biological model of disease translates into public health interventions that are based on a simplified logic, according to which to reduce the burden of disease it suffices to reduce exposure. However, health and disease are complex and this simplified logic often fails (Kelly & Russo, 2017). From the point of view of health care systems, in budgets across the high-income countries, this gives rise to the dominance of healthcare spending that aims to cure, rather than prevent, disease.

Our vision for change

Prioritizing interdisciplinarity interventions rather than exclusively biological interventions are especially relevant for *prevention, and to obtain sustainable effects of treatment*. Radically interdisciplinary knowledge should then be embedded into practice more widely, for instance in training of GPs, nurses, psychologists and health professionals. This embedding of humanities and social science approaches in public health programmes will gradually be

For peer review only - http://bmjopen.bmj.com/site/about/guidelines.xhtml

institutionalised. Existing best practices, such as TIP, should be exported widely. Yet exporting is hard: how do we know that something that worked once will work in a different context? External validity has been long recognised as crucial in scientific and policy settings (Cartwright & Hardie, 2012), and all the more so once the complexity of health and disease, due to psycho-socio-cultural and historical factors is brought to the fore. Answering this question requires local and global expertise across the micro-, meso- and macro-level of social organization. Practitioners make this engagement process possible, while emerging digital technologies can improve the participation of all actors involved.

Methodological

How things stand

One diagnosis for the lack of consideration of social and cultural factors in practice is the fragmentation of methods, as they are used in the health and social sciences. For example, obesity has biological, behavioural, psychological, and structural causes. Which methods should we use to understand each, and how should we combine them? Different interventions emphasise different causes – biological or social. We might identify structural (sugar tax), behavioural (food labelling), and biological (appetite-modifying drugs) approaches to prevent obesity (Hawkes et al., 2015; Jia et al., 2015; Kumanyika, Jeffery, Morabia, Ritenbaugh, & Antipatis, 2002). Yet the existence of different interventions does not produce a unified framework that addresses this plurality of causes. In fact, it is not a given that obesity reduces to biology, and therefore interventions that reflect the complexity of the phenomenon are much needed. Innovative biological approaches, such as precision medicine, fare no better.

Our vision for change

Our approach is *radical interdisciplinarity,* which places psycho-socio-cultural factors at the heart of health. We integrate methods and concepts used in the health sciences, in the humanities, and in the social sciences to understand how each influence and change our health and our understanding of health. The issue is not just to recognize *that* psycho-socio-cultural factors make a difference to health and disease, but *how and why* they are part of their aetiology. Asking normative questions about health and health interventions is integral to this approach. This will require reciprocal training for health scientists and practitioners, and those working in the medical humanities and social sciences. Only by joining forces we can achieve this radical interdisciplinarity and form knowledge that will ground a new generation of methods for studying health and for designing interventions.

For peer review only - http://bmjopen.bmj.com/site/about/guidelines.xhtml

Conceptual

How things stand

Just as methods in health are fragmented, so are concepts. We argue that the fragmentation of concepts in the study of health and disease results from the longstanding dominance of biological concepts (Cooper, 2016). But biology alone is insufficient to produce an adequate conceptualization of health. Similarly, adding a veneer of socio-cultural concern to a primarily biological foundation will, we think, not produce an adequate account of the complexity of health issues.

Our vision for change

Socio-psychological-cultural factors are active *causes*, that we argue should be understood on the same footing as biological causes (Kelly, Kelly, & Russo, 2014). The radical interdisciplinarity we advocate goes beyond embedding research from the social sciences and humanities into the existing health sciences. Scientific knowledge must be integrated with the lifeworld of individual patients, groups of patients, and of populations. This means having deep and detailed understanding of the conditions in which disease develop and evolves, including socio-economic factors, group dynamic, social support, access to health care infrastructure, etc. To achieve this, we advocate an exercise of thinking and working together to produce conceptual work about health in which different concepts are integrated together from the start: the social sciences and the medical humanities *together* with the health sciences can provide the concepts to account for the complexity of health and disease. This radical interdisciplinarity will produce understandable and practical concepts that can be used in research in the health sciences and in the humanities, and that can be integrated in the design of interventions, for the sake of health.

For peer review only

2. How to achieve this vision

At the *micro-level*, integrated approaches to this psycho-socio-cultural view of health require much more than awareness-raising. This may involve, for instance, changing the management of health so that the time factor in the relationship between the physician and the patient can be given more importance. We understand the urgent questions at this stage as less about ease of access, but quality of interaction, between health systems and healthy people.

For peer review only - http://bmjopen.bmj.com/site/about/guidelines.xhtml

At the *meso-level*, we need to develop measures that engender changes in civil society (families, school, churches, scout, unions). Direct promotion of solidarity, mutual help and healthy lifestyles is unlikely to be effective without careful contributions from the social sciences and humanities. Yet integrated social change can happen in civil society: initiatives to prevent substance abuse in Iceland, for example, were mediated by interventions that strengthen specific community-level factors influencing health (Sigfusdottir, Thorlindsson, Kristjansson, Roe, & Allegrante, 2008).

At the *macro-level*, structural interventions that involve transportation, taxation, and the fabric of government and commerce all promise potential benefits to health. We cannot hope to develop, evaluate, and implement these complex (and usually customized) works without analysing their effects across the many dimensions of practice and the social lifeworld.

Radical interdisciplinarity faces challenges at both the micro-, meso-, and macro-levels, and in the interactions of these levels[1]. We are aware of the scale of these challenges, but we are also confident that a new course in the way we study and intervene on health is achievable. This manifesto, a joint endeavour across the health sciences, public health, social sciences, and the humanities, is itself an exercise in radical interdisciplinarity.

Signatories

Claudi Bockting, Giovanni Boniolo, Stefano Canali, Brendan Clarke, Maria Conforti, O. Caglar Dede, Giulia Frezza, Mariacarla Gadebusch Bondio, Virginia Ghiara, Guido Giarelli, Phyllis Illari, Peng Jia, Michael P. Kelly, Louise Letley, Emiliano Mancini, Maria Marloth, Joko Mulyanto, Federica Russo, Maartje Schermer, Karien Stronks, Paolo Vineis.

References

Blue, S., Shove, E., Carmona, C., & Kelly, M. P. (2016). Theories of practice and public health: understanding (un)healthy practices. *Critical Public Health*, *26*(1), 36–50. https://doi.org/10.1080/09581596.2014.980396

[1] First successful attempts have been applied using an integrated approach. For example a dynamical systems approach to explain transmission of resistance in human immunodeficiency virus spreading and in-stent restenosis in coronary artery disease (Sloot & Hoekstra, 2010).

For peer review only - http://bmjopen.bmj.com/site/about/guidelines.xhtml

Braveman, P., Egerter, S., & Williams, D. R. (2011). The Social Determinants of Health: Coming of Age. *Annual Review of Public Health*, *32*(1), 381–398. https://doi.org/10.1146/annurev-publhealth-031210-101218

Cartwright, N., & Hardie, J. (2012). *Evidence-based policy: a practical guide to doing it better*. Oxford ; New York: Oxford University Press.

Commission on Social Determinants of Health. (2008). *Closing the gap in a generation: Health equity through action on the social determinants of health.* Geneva, Switzerland: World Health Organization.

Cooper, R. V. (2016). Health and disease. In J. A. Marcum (Ed.), *The Bloomsbury companion to contemporary philosophy of medicine* (pp. 275–296). London ; New York: Bloomsbury Academic, an imprint of Bloomsbury Publishing Plc.

Forman, D., Bauld, L., Bonanni, B., Brenner, H., Brown, K., Dillner, J., … Wild, C. P. (2018). Time for a European initiative for research to prevent cancer: A manifesto for Cancer Prevention Europe (CPE). *Journal of Cancer Policy*, *17*, 15–23. https://doi.org/10.1016/j.jcpo.2018.07.001

Friel, S., & Marmot, M. G. (2011). Action on the Social Determinants of Health and Health Inequities Goes Global. *Annual Review of Public Health*, *32*(1), 225–236. https://doi.org/10.1146/annurev-publhealth-031210-101220

Gibbon, S., Prainsack, B., Hilgartner, S., & Lamoreaux, J. (Eds.). (2018). *Handbook of genomics, health & society* (2nd ed.). Milton Park, Abingdon, Oxon ; New York, NY: Routledge.

Hawkes, C., Smith, T. G., Jewell, J., Wardle, J., Hammond, R. A., Friel, S., … Kain, J. (2015). Smart food policies for obesity prevention. *The Lancet*, *385*(9985), 2410–2421. https://doi.org/10.1016/S0140-6736(14)61745-1

Jia, P., Sankoh, O., & Tatem, A. J. (2015). Mapping the environmental and socioeconomic coverage of the INDEPTH international health and demographic surveillance system network. *Health & Place*, *36*, 88–96. https://doi.org/10.1016/j.healthplace.2015.09.009

Kelly, M. P., Kelly, R. S., & Russo, F. (2014). The Integration of Social, Behavioral, and Biological Mechanisms in Models of Pathogenesis. *Perspectives in Biology and Medicine*, *57*(3), 308–328. https://doi.org/10.1353/pbm.2014.0026

Kelly, M. P., & Russo, F. (2017). Causal narratives in public health: the difference between mechanisms of aetiology and mechanisms of prevention in non-communicable diseases. *Sociology of Health & Illness*, *40*(1), 82–99. https://doi.org/10.1111/1467-9566.12621

Kumanyika, S., Jeffery, R. W., Morabia, A., Ritenbaugh, C., & Antipatis, V. J. (2002). Obesity prevention: the case for action. *International Journal Of Obesity*, *26*, 425.

For peer review only - http://bmjopen.bmj.com/site/about/guidelines.xhtml

Marmot, M. (2005). Social determinants of health inequalities. *The Lancet*, *365*(9464), 1099–1104. https://doi.org/10.1016/S0140-6736(05)71146-6

Muntaner, C. (2013). Invited Commentary: On the Future of Social Epidemiology--A Case for Scientific Realism. *American Journal of Epidemiology*, *178*(6), 852–857. https://doi.org/10.1093/aje/kwt143

Napier, A. D., Ancarno, C., Butler, B., Calabrese, J., Chater, A., Chatterjee, H., … Woolf, K. (2014). Culture and health. *The Lancet*, *384*(9954), 1607–1639. https://doi.org/10.1016/S0140-6736(14)61603-2

Opolski, J. T., & Wysocki, M. J. (2013). "Health 2020"--new framework for health policy. Part II. *Przegl Epidemiol*, *67*(4), 647–650.

Sigfusdottir, I. D., Thorlindsson, T., Kristjansson, A. L., Roe, K. M., & Allegrante, J. P. (2008). Substance use prevention for adolescents: the Icelandic Model. *Health Promotion International*, *24*(1), 16–25. https://doi.org/10.1093/heapro/dan038

For peer review only

For peer review only - http://bmjopen.bmj.com/site/about/guidelines.xhtml

BMJ Open

Time to care: why the humanities and the social sciences belong in the science of health

Journal:	*BMJ Open*
Manuscript ID	bmjopen-2019-030286.R2
Article Type:	Communication
Date Submitted by the Author:	20-Jun-2019
Complete List of Authors:	Clarke, B; Imperial College London,<br>Ghiara, Virginia; University of Kent<br>Russo, Federica; Universiteit van Amsterdam,
**Primary Subject Heading**:	Public health
Secondary Subject Heading:	Epidemiology, Global health, Health policy, Qualitative research, Sociology
Keywords:	Medical humanities, Sociology of health, Prevention, Interdisciplinarity

SCHOLARONE™
Manuscripts

Time to care: why the humanities and the social sciences belong in the science of health

A. Authors: Brendan Clarke; Virginia Ghiara; Federica Russo (and co-signatories listed at the end of the article)
B. Corresponding author: Federica Russo, Department of Philosophy, University of Amsterdam, Postbus 94201, 1090 GE Amsterdam, Tel +31 (0)20 525 3537, email: f.russo@uva.nl / federica.russo@gmail.com
C. Co-authors: Brendan Clarke, University College London, London, UK; Virginia Ghiara, University of Kent, Canterbury, UK
D. Keywords: Medical humanities; Sociology of health; Prevention; Interdisciplinarity
E. Word count: 2043

Abstract
Health is *more* than the absence of disease. It is also more than a biological phenomenon. It is inherently social, psychological, cultural, and historical. While this has been recognised by major health actors for decades, open questions remain as to how to build systems that reflect the complexity of health, disease, and sickness, and in a context that is increasingly technologized. We argue that an urgent change of approach is necessary. Methods and concepts from the humanities and social science must be embedded in the concepts and methods of the health sciences if we are to promote sustainable interventions capable of engaging with the recognized complexity of health, disease, and sickness. Our vision is one of radical interdisciplinarity, integrating aspects of biological, psychological, social, and humanities approaches across areas of urgent health need. Radical interdisciplinarity, we argue, entails the practical, methodological, and conceptual integration of these approaches to health.

Signatories

This text is based on brain writing sessions in which all undersigned took part, during the Workshop "**Healthy People: How the Medical Humanities and the Health Sciences Study the Complexity of Health[1]**", held in Leiden between 29 October and 1 November

[1] https://www.lorentzcenter.nl/lc/web/2018/1089/info.php3?wsid=1089&venue=Snellius

For peer review only

2019, and supported by: NIAS-Lorentz Center, University of Amsterdam and AMC, University of Bonn, University College London.

Claudi Bockting[a], Giovanni Boniolo[b], Stefano Canali[c], Brendan Clarke[d], Maria Conforti[e], O. Caglar Dede[f], Giulia Frezza[e], Mariacarla Gadebusch Bondio[g], Virginia Ghiara[h], Guido Giarelli[i], Phyllis Illari[d], Peng Jia[l], Michael P. Kelly[m], Louise Letley[n], Emiliano Mancini[o], Maria Marloth[g], Joko Mulyanto[p], Federica Russo[q], Maartje Schermer[r], Karien Stronks[p], Paolo Vineis[s].

[a] Academic Medical Centre, Department of Psychiatry, University of Amsterdam, Amsterdam, Netherlands

[b] Dipartment of Biomedical Sciences, Università di Ferrara, Ferrara, Italy

[c] Institut für Philosophie, Leibniz University of Hannover, Hannover, Germany

[d] Department of Science and Technology Studies, University College London, London, United Kingdom

[e] Department of Molecular Medicine, University of Rome Sapienza, Rome, Italy

[f] Department of Philosophy, Erasmus University of Rotterdam, Rotterdam, Netherlands

[g] Institute for Medical Humanities, University of Bonn, Bonn, Germany

[h] Department of Philosophy, University of Kent, Canterbury, United Kingdom

[i] Faculty of Medicine, University of Catanzaro, Italy

[l] Department of Earth Observation Science, University of Twente, Enschede, Netherlands

[m] Institute of Public Health, Forvie Site, University of Cambridge, United Kingdom

[n] Immunisation, Hepatitis & Blood Safety Dept., National Infection Service, Public Health England, Wellington House, 133-155 Waterloo Road, London SE1 8UG, United Kingdom.

[o] Institute for Advanced Studies and Computational Science Laboratory, University of Amsterdam, Amsterdam, Netherlands

[p] Department of Public Health, Academic Medical Center, University of Amsterdam, Amsterdam, Netherlands

[q] Department of Philosophy, University of Amsterdam, Amsterdam, Netherlands

[r] Department of Medical Ethics and Philosophy of Medicine, Erasmus MC, Rotterdam, The Netherlands

[s] School of Public Health, Department of Epidemiology and Biostatistics, Imperial College London, London, United Kingdom

1. Our vision

For peer review only - http://bmjopen.bmj.com/site/about/guidelines.xhtml

Health is *more* than the absence of disease. It is also more than a biological phenomenon. It is inherently social, psychological, cultural, and historical. Social and personal resources are both key components and key determinants of health, as it has been recognised by major health actors for decades [1–3]. However, open questions remain as to how to build systems that reflect the complexity of health, healthy lives, disease, and sickness, and in a context that is increasingly technologized. Although we find in the literature an increasing understanding of the complexity of health [4–7], the implementation of this knowledge lags behind. Biological approaches to health and disease, as a matter of fact, dominate the development of curative and preventive interventions.

We argue that an urgent change of approach is necessary. Methods and concepts from the humanities and social science must be *embedded* in the concepts and methods of the health sciences and of public health, if we are to promote sustainable interventions capable of engaging with the recognized complexity of health, healthy lives, disease, and sickness. This resonates with the vision expressed by UK Health Secretary and by many policy documents [8,9] from the last decades. Yet, given the difficulties associated with interdisciplinary research, integrated strategies to understand and to intervene on the complexity of health and that engage with biological, social, psychological and behavioural factors are still needed.

Our vision is one of *radical interdisciplinarity*, integrating aspects of biological, psychological, social, and humanities approaches across areas of urgent health need. These areas include, but is not confined to, chronic conditions such as the obesity epidemic, cancer, mental health. Radical interdisciplinarity entails the **practical**, **methodological**, and **conceptual** integration of approaches to health, as they are developed in the health and social sciences, and in the humanities. It is the combination of cognitive resources from individuals belonging to different disciplines, who accept and respect the division of labour and the resulting epistemic dependence to tackle phenomena that would not be adequately conceptualised within any of the involved discipline alone [10]. In what follows, we describe our current understanding of these three aspects, and describe how radical interdisciplinarity would change them.

Practical

How things stand

The impact of social factors on health is widely acknowledged in the scientific literature [11–15]. However, this does not always mean that they are effectively actioned in health care

For peer review only - http://bmjopen.bmj.com/site/about/guidelines.xhtml

and public health. Best practices do exist, but they do not constitute, as yet, the dominant approach. We think that exemplary public health programmes, such as *WHO Tailoring Immunization Programmes (TIP)*, are strengthened and effective precisely because they explicitly target social factors and exploit deep knowledge of cultural factors and social dynamic in the design of interventions. Despite the successes of interventions targeting social and cultural factors, more distinctively biological models of health predominate in practice [9]. The dominant biological model of disease translates into public health interventions that are based on a simplified logic, according to which to reduce the burden of disease it suffices to reduce exposure. This idea, furthermore, often involves mere behavioural changes at the individual level. While this simplified logic allows for clear, unequivocal criteria of success and avoids complex and difficult discussions about responsibilities at the structural level, it often fails [16]. From the point of view of health care systems, in budgets across the high-income countries, this gives rise to the dominance of healthcare spending that aims to cure, rather than prevent, disease. This calls for a deep and interdisciplinary reflection also on what 'care' means – the work of e.g. Mol [17] and Mol, Mosel, Pols [18] are good examples of how this can be done.

Our vision for change

Prioritizing interdisciplinarity interventions rather than exclusively biological interventions are especially relevant for *prevention, and to obtain sustainable effects of treatment*. Radically interdisciplinary knowledge should then be embedded into practice more widely, for instance in training of GPs, nurses, psychologists and health professionals. This embedding of humanities and social science approaches in public health programmes will gradually be institutionalised and will help to solve the major challenges of interdisciplinarity, such as epistemic dependence, the lack of a shared vocabulary and of a mutual recognition of the role played by the other disciplines [10,19]. Existing best practices, such as the 'Tailoring Immunisation programmes[2]' (TIP), should be exported widely. Yet exporting is hard: how do we know that something that worked once will work in a different context? External validity has been long recognised as crucial in scientific and policy settings [20], and all the more so once the complexity of health and disease, due to psycho-socio-cultural and historical factors is brought to the fore. Answering this question requires local and global expertise across the different levels of social organizations. Practitioners make this engagement process possible,
while emerging digital technologies can provide new opportunities to involve, as active partners, communities and patients with relevant health experiences.

Methodological

[2] https://www.who.int/immunization/programmes_systems/Global_TIP_overview_July2018.pdf?ua=1

For peer review only - http://bmjopen.bmj.com/site/about/guidelines.xhtml

How things stand

One diagnosis for the lack of consideration of social and cultural factors in practice is the fragmentation of methods, as they are used in the health and social sciences. While quantitative methods can be used to effectively study certain health dimensions, psychological and cultural aspects can often be understood only by means of qualitative approaches. How to integrate different epistemological and methodological perspectives, however, is in general not straightforward. For example, obesity has biological, behavioural, psychological, environmental, and structural causes [21,22]. Which methods should we use to understand each, and how should we combine them? Different interventions emphasise different causes – biological or social. We might identify structural (sugar tax), environmental (walkable neighbourhoods), behavioural (food labelling), and biological (appetite-modifying drugs) approaches to prevent obesity [23–25]. Yet the existence of different interventions does not produce a unified framework that addresses this plurality of causes, and does not solve the tension between individual and structural responsibilities [26–28]. In addition, innovative biological approaches, such as precision medicine and emerging technologies are often used as 'magnifier' to see better into 'the biology' [29,30]. Such approaches run the risk of not giving due importance to social and cultural factors influencing patients' life and health.

Our vision for change

Our plea is for *radical interdisciplinarity,* which places psycho-socio-cultural factors at the heart of health. We integrate methods and concepts used in the health sciences, in the humanities, and in the social sciences to understand how each influence and change our health and our understanding of health. The issue is not just to recognize *that* psycho-socio-cultural factors make a difference to health and disease, but *how and why* they are part of their aetiology, of concept of health itself, and of different ways of understanding 'care'. Asking normative questions about health, health interventions, and health data use is integral to this approach. This will require reciprocal training for health scientists and practitioners, and those working in the medical humanities and social sciences. In our view, mixed methods research has positively contributed to investigating the complexity of health-related phenomena in an effective way [31,32]; we think that this strategy is likely to be the most suitable to explore the different dimensions of health. Similarly, more integrative methodological approaches are better suited to assess complex dimensions of health interventions such as their impact on health inequalities (Dede, 2019). To this end, it is useful to think about networks of various kinds of health scientists and how their different methodological practices are integrated. Only by joining forces we can achieve this radical

For peer review only - http://bmjopen.bmj.com/site/about/guidelines.xhtml

interdisciplinarity and form knowledge that will ground a new generation of methods for studying health and for designing interventions.

Conceptual

How things stand

Just as methods in health are fragmented, so are concepts. We argue that the fragmentation of concepts in the study of health and disease results from the longstanding dominance of biological concepts [33]. But biology alone is insufficient to produce an adequate conceptualization of health. Similarly, adding a veneer of socio-cultural concern to a primarily biological foundation will, we think, not produce an adequate account of the complexity of health issues.

Our vision for change

Socio-psychological-cultural factors are vital health dimensions and active health *causes*, that we argue should be understood on the same footing as biological causes [34]. The radical interdisciplinarity we advocate goes beyond embedding research from the social sciences and humanities into the existing health sciences. Scientific knowledge must be integrated with the lifeworld of individual patients, groups of patients, and of populations. This means having deep and detailed understanding of the conditions in which disease develop and evolves, including socio-economic factors, group dynamic, social support, access to health care infrastructure, etc. To achieve this, we advocate an exercise of thinking and working together to produce conceptual work about health in which different concepts are integrated together from the start: the social sciences and the medical humanities *together* with the health sciences can provide the concepts to account for the complexity of health and disease. This radical interdisciplinarity will produce understandable and practical concepts that can be used in research in the health sciences and in the humanities, and that can be integrated in the design of interventions, for the sake of health.

2. How to achieve this vision

Researchers in the health and social sciences and in the humanities, practitioners, and policy makers need to collaborate with a variety of networks of health actors.

To promote health across individuals, integrated approaches to this psycho-socio-cultural view of health require much more than awareness-raising. This may involve, for instance,

For peer review only - http://bmjopen.bmj.com/site/about/guidelines.xhtml

changing the management of health so that the time factor in the relationship between the physician and the patient can be given more importance. We understand the urgent questions at this stage as less about ease of access, but quality of interaction, between health systems and healthy people.

Given the social and group dimension of health, we need to develop measures that engender changes in civil society (families, school, churches, scout, unions). Direct promotion of solidarity, mutual help and healthy lifestyles is unlikely to be effective without careful contributions from the social sciences and humanities. Yet integrated social change can happen in civil society: initiatives to prevent substance abuse in Iceland, for example, were mediated by interventions that strengthen specific community-level factors influencing health, including the creation of a parental network, the promotion of community activities such as parental prowl (parents walking around their neighbourhood together during weekend nights) and the availability of organized youth activities [35]).

Finally, at the broader level of population, structural interventions that involve transportation, taxation, and the fabric of government and commerce all promise potential benefits to health. We cannot hope to develop, evaluate, and implement these complex (and usually customized) works without analysing their effects across the many dimensions of practice and the social lifeworld.

Radical interdisciplinarity faces challenges at all these levels [3]. We are aware of the scale of these challenges, but we are also confident that a new course in the way we study and intervene on health is achievable. This manifesto, a joint endeavour across the health sciences, public health, social sciences, and the humanities, is itself an exercise in radical interdisciplinarity, based on combined efforts to create a common ground for the future of research on health and disease, and for design and implementation interventions that can drive health care systems towards health.

[3] First successful attempts have been applied using an integrated approach. For example a dynamical systems approach to explain transmission of resistance in human immunodeficiency virus spreading and in-stent restenosis in coronary artery disease (Sloot & Hoekstra, 2010).

For peer review only - http://bmjopen.bmj.com/site/about/guidelines.xhtml

Contributorship statement

B. Clarke, V. Ghiara, and F. Russo drafted a full text on the basis of brain writing sessions which all signatories of this communication article took part in during the workshop "Healthy People: How the Medical Humanities and the Health Sciences Study the Complexity of Health", held in Leiden between 29 October and 1 November 2019.

Competing interests

All authors declare no financial relationships with any organisations that might have an interest in the submitted work; no other relationships or activities that could appear to have influenced the submitted work.

Funding

We gratefully acknowledge funding from the NIAS-Lorentz Center, University of Amsterdam and AMC, University of Bonn, University College London that made possible the organization of the workshop "**Healthy People: How the Medical Humanities and the Health Sciences Study the Complexity of Health"**.

For peer review only

References

1 Hinchliffe S, Jackson MA, Wyatt K, *et al.* Healthy publics: enabling cultures and environments for health. *Palgrave Commun* 2018;**4**. doi:10.1057/s41599-018-0113-9

2 Huber M, Knottnerus JA, Green L, *et al.* How should we define health? *BMJ* 2011;**343**:d4163–d4163. doi:10.1136/bmj.d4163

3 WHO. Constitution of the World Health Organization. 2006. https://www.who.int/governance/eb/who_constitution_en.pdf

4 Gibbon S, Prainsack B, Hilgartner S, *et al.*, editors. *Handbook of genomics, health & society*. 2nd ed. Milton Park, Abingdon, Oxon ; New York, NY: : Routledge 2018.

5 Friel S, Marmot MG. Action on the Social Determinants of Health and Health Inequities Goes Global. *Annu Rev Public Health* 2011;**32**:225–36. doi:10.1146/annurev-publhealth-031210-101220

6 Opolski JT, Wysocki MJ. "Health 2020"--new framework for health policy. Part II. *Przegl Epidemiol* 2013;**67**:647–50.

7 Blue S, Shove E, Carmona C, *et al.* Theories of practice and public health: understanding (un)healthy practices. *Crit Public Health* 2016;**26**:36–50. doi:10.1080/09581596.2014.980396

8 Forman D, Bauld L, Bonanni B, *et al.* Time for a European initiative for research to prevent cancer: A manifesto for Cancer Prevention Europe (CPE). *J Cancer Policy* 2018;**17**:15–23. doi:10.1016/j.jcpo.2018.07.001

9 Napier AD, Ancarno C, Butler B, *et al.* Culture and health. *The Lancet* 2014;**384**:1607–39. doi:10.1016/S0140-6736(14)61603-2

10 Andersen H. Collaboration, interdisciplinarity, and the epistemology of contemporary science. *Stud Hist Philos Sci Part A* 2016;**56**:1–10. doi:10.1016/j.shpsa.2015.10.006

11 Braveman P, Egerter S, Williams DR. The Social Determinants of Health: Coming of Age. *Annu Rev Public Health* 2011;**32**:381–98. doi:10.1146/annurev-publhealth-031210-101218

12 Commission on Social Determinants of Health. Closing the gap in a generation: Health equity through action on the social determinants of health. Geneva, Switzerland: : World Health Organization 2008.

13 Jia P, Sankoh O, Tatem AJ. Mapping the environmental and socioeconomic coverage of the INDEPTH international health and demographic surveillance system network. *Health Place* 2015;**36**:88–96. doi:10.1016/j.healthplace.2015.09.009

14 Marmot M. Social determinants of health inequalities. *The Lancet* 2005;**365**:1099–104. doi:10.1016/S0140-6736(05)71146-6

15 Muntaner C. Invited Commentary: On the Future of Social Epidemiology--A Case for Scientific Realism. *Am J Epidemiol* 2013;**178**:852–7. doi:10.1093/aje/kwt143

16 Kelly MP, Russo F. Causal narratives in public health: the difference between mechanisms of aetiology and mechanisms of prevention in non-communicable diseases. *Sociol Health Illn* 2017;**40**:82–99. doi:10.1111/1467-9566.12621

17 Mol A. *The logic of care: health and the problem of patient choice*. London ; New York: : Routledge 2008.

18 Mol A, Moser I, Pols J, editors. *Care in practice: on tinkering in clinics, homes and farms*. 1. Aufl. Bielefeld: : Transcript-Verl 2010.

19 Callard F, Fitzgerald D. *Rethinking interdisciplinarity across the social sciences and neurosciences*. Basingstoke, Hampshire New York, NY: : Palgrave Macmillan 2015.

20 Cartwright N, Hardie J. *Evidence-based policy: a practical guide to doing it better*. Oxford ; New York: : Oxford University Press 2012.

21 Jia P, Xue H, Cheng X, *et al.* Effects of school neighborhood food environments on childhood obesity at multiple scales: a longitudinal kindergarten cohort study in the USA. *BMC Med* 2019;**17**. doi:10.1186/s12916-019-1329-2

22 Jia P, Xue H, Cheng X, *et al.* Association of neighborhood built environments with childhood obesity: Evidence from a 9-year longitudinal, nationally representative survey in the US. *Environ Int* 2019;**128**:158–64. doi:10.1016/j.envint.2019.03.067

For peer review only

For peer review only - http://bmjopen.bmj.com/site/about/guidelines.xhtml

23 Hawkes C, Smith TG, Jewell J, *et al.* Smart food policies for obesity prevention. *The Lancet* 2015;**385**:2410–21. doi:10.1016/S0140-6736(14)61745-1

24 Kumanyika S, Jeffery RW, Morabia A, *et al.* Obesity prevention: the case for action. *Int J Obes* 2002;**26**:425.

25 Wang Y, Jia P, Cheng X, *et al.* Improvement in food environments may help prevent childhood obesity: Evidence from a 9-year cohort study. *Pediatr Obes* 2019;:e12536. doi:10.1111/ijpo.12536

26 Powers M, Faden RR. *Social justice: the moral foundations of public health and health policy*. Oxford: : Oxford Univ. Press 2008.

27 Stol YH, Schermer MHN, Asscher ECA. Omnipresent Health Checks May Result in Over-responsibilization. *Public Health Ethics* 2016;:phw034. doi:10.1093/phe/phw034

28 Venkatapuram S. *Health justice: an argument from the capabilities approach*. Cambridge Malden, MA: : Polity 2011.

29 Jia P. Spatial lifecourse epidemiology. *Lancet Planet Health* 2019;**3**:e57–9. doi:10.1016/S2542-5196(18)30245-6

30 Saracci R. Epidemiology in wonderland: Big Data and precision medicine. *Eur J Epidemiol* 2018;**33**:245–57. doi:10.1007/s10654-018-0385-9

31 Greene JC. Preserving Distinctions Within the Multimethod and Mixed Methods Research Merger. In: Hesse-Biber SN, Johnson RB, eds. *The Oxford Handbook of Multimethod and Mixed Methods Research Inquiry*. Oxford, United Kingdom: : Oxford University Press 2015. 606–15.

32 Tariq S, Woodman J. Using mixed methods in health research. *JRSM Short Rep* 2013;**4**:204253331347919. doi:10.1177/2042533313479197

33 Cooper RV. Health and disease. In: Marcum JA, ed. *The Bloomsbury companion to contemporary philosophy of medicine*. London ; New York: : Bloomsbury Academic, an imprint of Bloomsbury Publishing Plc 2016. 275–96.

34 Kelly MP, Kelly RS, Russo F. The Integration of Social, Behavioral, and Biological Mechanisms in Models of Pathogenesis. *Perspect Biol Med* 2014;**57**:308–28. doi:10.1353/pbm.2014.0026

35 Sigfusdottir ID, Thorlindsson T, Kristjansson AL, *et al.* Substance use prevention for adolescents: the Icelandic Model. *Health Promot Int* 2008;**24**:16–25. doi:10.1093/heapro/dan038

For peer review only
